# Under-Detection of Lyme Disease in Canada

**DOI:** 10.3390/healthcare6040125

**Published:** 2018-10-15

**Authors:** Vett K. Lloyd, Ralph G. Hawkins

**Affiliations:** 1 Department Biology, Mt. Allison University, Sackville, NB E4L 1E2, Canada; 2 Division of General Internal Medicine, University of Calgary, South Health Campus, Calgary, AB T3M 1M4, Canada; rghawkin@ucalgary.ca

**Keywords:** Lyme disease, Canada, ticks, C6 ELISA, WCS ELISA, immunoblotting, canine seroprevalence, tick surveillance, Lyme disease prevalence, Lyme disease incidence

## Abstract

Lyme disease arises from infection with pathogenic *Borrelia* species. In Canada, current case definition for confirmed Lyme disease requires serological confirmation by both a positive first tier ELISA and confirmatory second tier immunoblot (western blot). For surveillance and research initiatives, this requirement is intentionally conservative to exclude false positive results. Consequently, this approach is prone to false negative results that lead to underestimation of the number of people with Lyme disease. The province of New Brunswick (NB), Canada, can be used to quantify under-detection of the disease as three independent data sets are available to generate an estimate of the true human disease prevalence and incidence. First, detailed human disease incidence is available for the US states and counties bordering Canada, which can be compared with Canadian disease incidence. Second, published national serology results and well-described sensitivity and specificity values for these tests are available and deductive reasoning can be used to query for discrepancies. Third, high-density tick and canine surveillance data are available for the province, which can be used to predict expected human Lyme prevalence. Comparison of cross-border disease incidence suggests a minimum of 10.2 to 28-fold under-detection of Lyme disease (3.6% to 9.8% cases detected). Analysis of serological testing predicts the surveillance criteria generate 10.4-fold under-diagnosis (9.6% cases detected) in New Brunswick for 2014 due to serology alone. Calculation of expected human Lyme disease cases based on tick and canine infections in New Brunswick indicates a minimum of 12.1 to 58.2-fold underestimation (1.7% to 8.3% cases detected). All of these considerations apply generally across the country and strongly suggest that public health information is significantly under-detecting and under-reporting human Lyme cases across Canada. Causes of the discrepancies between reported cases and predicted actual cases may include undetected genetic diversity of *Borrelia* in Canada leading to failed serological detection of infection, failure to consider and initiate serological testing of patients, and failure to report clinically diagnosed acute cases. As these surveillance criteria are used to inform clinical and public health decisions, this under-detection will impact diagnosis and treatment of Canadian Lyme disease patients.

## 1. Introduction

Lyme disease, or Lyme borreliosis, is a serious disease resulting from infection with *Borrelia* species of the Lyme borreliosis group, formerly known as *Borrelia burgdorferi* sensu lato [1,2]. Untreated and undertreated infections cause debilitating and sometimes fatal multi-system organ malfunction in humans and companion animals [1,3,4,5,6,7,8,9,10,11,12]. The primary vectors are Ixodid ticks, in eastern and central Canada including the prairie provinces (Manitoba, Saskatchewan, and Alberta) *Ixodes scapularis* and in British Columbia Canada mainly, *Ixodes pacificus*. The ticks are both introduced by migratory animals such as birds [1,13,14,15,16] and arise from locally established, breeding (endemic) tick populations [1,17,18].

In Canada, a confirmed diagnosis of disseminated (non-acute Lyme disease in which the bacteria have disseminated through the body from the site of initial infection, as defined by the Public Health Agency of Canada [19]) Lyme disease requires positive laboratory evidence, usually two-tiered serology, whereas a probable case requires physician-documented erythema migrans rash and exposure to a designated endemic site [19]. Thus, in order for a human Lyme disease case to be recorded, a Lyme diagnosis must be first considered by both patient and health care provider. For a confirmed case, serology must be then initiated and both tiers of the serology must be positive, while for probable cases the diagnosis, once made, must be reported. This chain of requirements is intended to generate unambiguous data for surveillance purposes and is intentionally conservative for that reason. But because of the complex interplay between the health care system, biology, and diagnostics, this approach under-detects and under-reports the true prevalence and incidence of human *Borrelia* infections.

Surveillance information is intertwined with clinical disease management as well as public health and societal responses. Clinical management of Lyme disease is significantly impacted by disease surveillance data. While formally a clinical diagnosis, case definition permitting clinical diagnosis of acute Lyme disease depends upon exposure to a Lyme disease risk area, which is in part informed by estimates of disease prevalence [19], so reluctance to provide a clinical diagnosis when these conditions are not met is understandable. Public health assessment of the practices of family physicians in both Quebec and New Brunswick have noted this reluctance and a strong reliance on serology, particularly in areas of emerging risk [20,21]. Similarly, public health initiatives and public policy responses are triggered when the incidence of disease exceed certain thresholds. Underestimation of *Borrelia* infection incidence has been explored in the United States where the United States Centers for Disease Control and Prevention (CDC) recently increased their estimates of the number of Lyme cases by approximately 10-fold. Hinckley et al. (2014) [22] evaluated the results of testing, including conventional two-tiered serological testing for Lyme disease in seven large commercial laboratories, and documented 21.3 to 39.4-fold under-reporting of Lyme cases. Similarly, Nelson et al. (2015) [23] documented 11.5 to 14.6-fold under-reporting of Lyme disease to the CDC based on national health insurance claim coding and treatment regimens.

There is general acceptance that, while mandatory, reporting in Canada underestimates the incidence (number of individuals in a given period who become newly infected) and prevalence (the number of individuals presently afflicted, which is the incidence multiplied by the average duration of the disease) in a population [24,25,26,27]. However, no analysis to estimate the magnitude of under-detection in Canada has been undertaken. The province of New Brunswick (NB) provides an excellent opportunity for this analysis because the province shares a similar climate, wildlife, and extensive border with the state of Maine, ME, USA [28,29], and surveillance information is available from both regions [30,31]. Additionally, human serology results including the number of human Lyme disease cases that meet surveillance definitions have been published for the province [32,33], which provide an additional means to assess disease detection efficiency. Finally, longitudinal region-specific tick surveillance data, which includes *Borrelia* infection prevalence [34,35,36], data on tick feeding time from various hosts [34,35,36], and region-specific canine *Borrelia* seroprevalence data [37,38,39] are all available for the province. New Brunswick is a province on the eastern coast of Canada, bordered by the Canadian provinces of Nova Scotia and Quebec, the American state of Maine, and the Atlantic Ocean. In 2014, the time period during which the surveillance information being analyzed here was obtained, there were two known endemic tick populations in the southwest of the province [40]. The province is at the intersection of the North American Atlantic and the trans-Atlantic eastern migratory bird flyways, an important source for the introduction of ticks [13,41], and is at the leading edge of invasion and establishment of permanent populations of the *Borrelia* vector, *Ixodes scapularis*, the black-legged tick [18,30].

Analysis of these independent data sets collectively allows three independent estimates of the extent to which the public health surveillance data under-detects the prevalence and incidence of Lyme disease in the Canadian population. This work indicates areas where further research and information capture is required for better risk estimates, both in areas of emerging risk and in parts of the country where endemic tick populations are widespread. This work demonstrates that tick vectored diseases pose a significant, under-detected and under-reported public health challenge in Canada.

## 2. Materials and Methods

### 2.1. Source Data

The analyses reported here rely on data in major public health databases and published, peer-reviewed scientific literature. All available Canadian serological studies were included. Translation of canine seroprevalence to human Lyme borreliosis is based on tick feeding time (**F**), multiplied by tick infection (**I**), multiplied by the number of tick bites (**T**), as detailed in Section 3.3. Each of these values relies on data drawn from different studies, each of which has its own inherent limitations, so we chose studies with the largest or most robust data sets and if there was a discrepancy, used the most conservative values. Tick infection values (**I**) vary slightly depending on the population of ticks assessed, their origin, the sample size, and possibly method of tick testing. The national tick surveillance program [35] reports 6.8% of NB ticks carried *Borrelia burgdorferi* in 2012 and Ogden et al. (2006) [34] reported an infection rate of 15.9% from 1990–2003. The value used here, 12.3%, is from the most recent, and highest density study (*n* = 536) [36] and is bracketed by these values. Estimation of tick feeding time was derived from tick collection data as described in Section 2.2, below. Regrettably, tick engorgement is not regularly quantified; Dibernardo et al. (2014) [35] provide data for ticks derived from NB human hosts (*n* = 123), but no quantitative indication of the extent of engorgement was provided. For determining the number of canine tick bites, Bjurman et al. (2016) [37] report 6% of NB dogs were seropositive in 2013/2014 (*n* = 699). There are two other studies reporting seropositivity for dogs in NB. Herrin et al. (2017) [38] report 3.7% canine seropositivity for NB dogs, however, their data does not include the western third of the province that incorporates the known endemic sites and the regions abutting the Maine border. Evason (2017) [39], using a larger sample size than used in Bjurman et al. (2016) [37] provides a preliminary report giving 6.7% and 8.8% seroprevalence in NB dogs in 2008 and 2015, respectively. Both of these values are higher than the 6% value used here suggesting that this value is conservative.

### 2.2. Estimation of Tick Feeding Time

Estimation of the duration of feeding of adult female *I. scapularis* ticks based on body size was performed following the approach of Gray et al. (2005) [42]. Dorsal measurements of female adult tick, body and scutum length and width, were taken and the body length/scutum width ratio (parameters chosen to minimize distortion due to damaged or dried specimens) were anchored against values from Gray et al. (2005) [42] in which these measurements were calculated for *I. ricinus* after defined feeding times. For *I. scapularis* recovered from canine hosts, ticks recovered in 2014 from the Mount Allison tick bank were used (*n* = 348), and for ticks recovered from human hosts, *I. scapularis* from 2012–2016 Mount Allison tick bank submissions (*n* = 194) were used in order to increase sample size. Collection of tick specimens in the Mount Allison tick bank has been approved by the Mount Allison Animal Care Committee as necropsy/invertebrate approval 101,971.

### 2.3. Statistics

The results of predicted serological result and those observed were tested with Fisher’s Exact Test.

## 3. Results

### 3.1. Comparison of Lyme Disease Incidence in Canada and the United States

The incidence (per 100,000) of Lyme disease in Canadian provinces for which data is publicly available for both 2014 and 2016, and incidence in the bordering or nearest American states, are shown in Table 1. The difference in the incidences between the two countries decreased between 2014 and 2016, depending on the region and year, suggests that increased physician and public awareness, among other factors, is decreasing under-detection. Nevertheless, there is a 2.5 to 133-fold difference, in the incidence of Lyme disease reported between neighboring regions of the two countries, corresponding to 0.8% to 40% lower detection in Canada. This difference, demarked by a political border that does not influence the biology of ticks or their hosts, strongly suggests impaired detection of Lyme disease in Canada, particularly as the US incidences are not corrected for the documented under-reporting [22,23]. This under-reporting would not be expected to affect the Canadian confirmed Lyme disease incidence metrics as both the serological testing required for a confirmed diagnosis and the collection of surveillance information is centered within the Public Health Agency of Canada.

A potential cause for the difference in incidence between countries is the well-characterized North-South and coastal-inland cline in tick abundance [43]. To assess the difference in disease incidence across the border that could be attributed solely to geographic causes, we focused on reported Lyme disease incidences between Maine, ME, USA and NB, Canada. The state of Maine, USA reported 1169 confirmed Lyme cases in 2014, giving an incidence of 87.9 per 100,000 [31]. During the same year, NB reported 5 cases [32], giving an incidence of 0.66 and a 133-fold difference between the two regions. This difference is notable given the extensive border, similar climate, and wild *B. burgdorferi* reservoir-competent species [28,29]. However, if only cases from two counties bordering NB, Aroostook, and Washington are considered, the number of cases is then 19 [31], giving an incidence of 18.4, only 28-fold higher than the NB incidence. Further, if the NB cases are assumed to have occurred in the county containing the two identified endemic areas and/or in the counties that abut the Maine border, this generates an incidence of 1.8, 10.2 times lower (9.8%) than the incidence across the border. The American and Canadian counties flanking the border are separated by approximately 100 km. Ecological differences within that scale are unlikely to account for this discrepancy suggesting failure to efficiently identify Lyme disease cases in NB, and similarly throughout Canada, is due to detection and reporting processes specific to Canada.

### 3.2. Assessment of Serological Diagnostics

Both diagnosis and surveillance classification of disseminated Lyme disease are dominantly dependent on serological testing. Diagnosis of Lyme disease can be based on clinical criteria alone in the acute phase, but for early and late disseminated stages the case definitions require laboratory confirmation [19]. Similarly for surveillance, a “confirmed” disease is defined as any case with clinical evidence of infection and laboratory confirmation and a “probable” case requires laboratory evidence of infection for disseminated stages although acute disease can be recorded without laboratory confirmation if other conditions are met. As laboratory evidence predominantly relies on serological testing, this means that published serological data can be used to estimate the magnitude of both prevalence and incidence. While there is a paucity of seroprevalence studies involving Lyme disease in Canadian populations, one such study in the province of Nova Scotia has been published [44].

**Figure 1:**
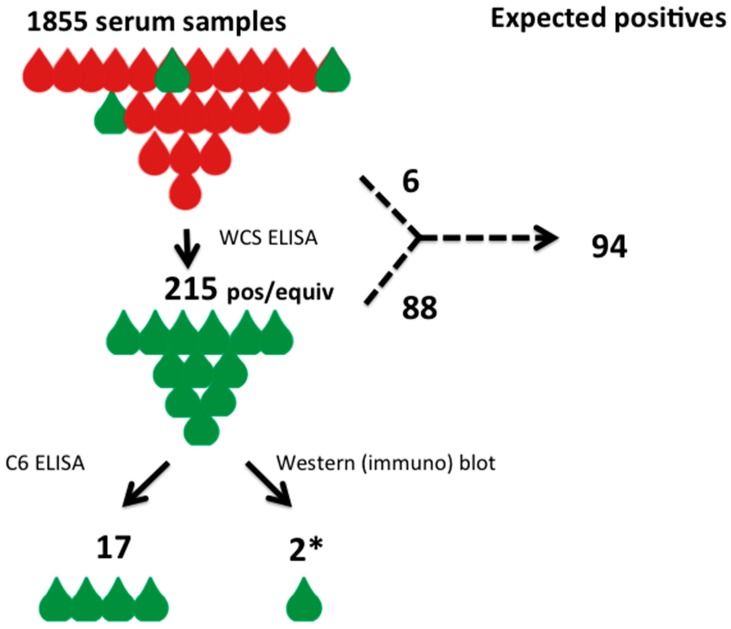
A schematic diagram of the seroprevalence study of Hatchette et al. (2015) [35] indicating observed results and the results that would be expected given the known sensitivity and specificity of the WCS ELISA. Red drops indicate non-reactive serum samples, green drops indicate those with evidence of anti-*Borrelia* antibodies. The study data is cartooned on the left with the tests performed and the observed results indicated. The expected results, based on known test characteristics, is shown on the right. The asterisk indicates that the 2 positive western blot samples were only positive using relaxed CDC criteria.

In the study, an anonymous sampling of 1855 remnant serum specimens from various health regions of Nova Scotia were analyzed for evidence of *Borrelia burgdorferi* seromarkers (Figure 1) [44]. The anonymous sera used in this study was derived from patients who had blood testing initiated as part of their other regular medical care. No information on clinical suspicion of *Borrelia* infection is available and although the entire province of Nova Scotia is currently considered either medium or high risk for Lyme disease with endemic areas increasing in number and size, this may not have been the case when the samples were drawn. The initial analysis was done using a commercial whole-cell sonicate ELISA assay (WCS ELISA), and identified 215 positive or equivocal results in the 1855 specimens tested (11.6%). Two secondary analyses were then done on the 215 positive screened specimens; a C6 ELISA assay was employed as a confirmatory test and, in parallel, all 215 specimens were subjected to western blot analysis using the standard CDC seropositivity definition. The C6 ELISA assay identified 17 specimens that were positive, meaning that amongst the 1855 initial specimens tested, 17 fulfilled one proposed criteria for seropositivity (Figure 1) [45]. In contrast, western blot analysis detected none that were positive by CDC criteria, although the investigators permitted, for the sake of the seroprevalence study, a “borderline” positive definition for two specimens with 4 rather than 5 western blot bands (Figure 1) [44].

The only test applied to the 1855 group specimen at large was the WCS ELISA test. This test is well characterized and its sensitivity and specificity parameters have been published previously [46,47]. This permits the data to be evaluated using deductive reasoning [48], where the test outcome can be anticipated based upon the known parameters of the technology being applied. Of the total 1855 specimens, 215 tested positive or equivocal. The WCS ELISA employed in the study has a known sensitivity of 93.3% and known specificity of 92.6% for later stage Lyme disease detection (sensitivity is lower in early stage disease, however serological testing is not recommended for acute disease [19] and would have been unlikely to have been included by chance alone, so the more conservative approach of using late stage parameters, hence predicting fewer undetected cases, has been used here). Based on the observed test results and the positive and negative predictive values, a two-way table can be constructed to deduce the true seropositivity value for the population from which the generated results were derived (Table 2). This analysis, based on known test characteristics, identifies that Lyme disease seroprevalence was identified by the WCS ELISA in the target population in 88 individuals, with 127 false-positive results, and a diagnosis would have been missed by the assay in 6 individuals for an overall prevalence of 94/1855 (5.1%).

The secondary testing using the C6 ELISA assay identified 17 seropositive individuals (prevalence 0.92%); and the western blot failed to identify any seropositive individuals using established CDC reporting criteria, although “borderline” positive results were identified in 2 specimens (prevalence 0.1%) using relaxed criteria. These results are all significantly divergent from each other using Fisher’s Exact Test (2-ELISA method vs. western blot *p* = 0.0006; 2-ELISA method vs. Deductive analysis *p* < 0.00001; western blot vs. Deductive analysis *p* < 0.00001).

The only test applied to all of the 1855 specimens was the WCS ELISA, and therefore one could argue that only the results of the WCS ELISA should be engaged to define the seroprevalence in the larger group tested rather than the secondary tests applied only to a subset. The deductive analysis identifies an inferential seroprevalence that is more than 5-fold (94 expected positives versus the 17 observed) greater in this Nova Scotia population than is identified by the two-ELISA method, and 47-fold (94/2) higher seroprevalence than identified using standard ELISA and western blot two-tier testing, even with a relaxed variation of western blot criteria. The two-ELISA method identifies 8.5-fold (17/2) higher seroprevalence than the two-tier methodology. Disturbingly, none of the 1855 specimens met the defined CDC seroprevalence criteria, and would still be formally considered as ‘Negative’ in the setting of a clinical presentation, highlighting its lack of reliable sensitivity for its use for seroprevalence surveillance. If the CDC criteria were to be rigidly applied in this circumstance as defined for serodiagnostic purposes, all 215 WCS ELISA positive tests and all 17 of the double ELISA tests would be deemed to be “false-positives”. For either of the lower seroprevalence estimates derived from secondary testing to be accepted as valid, there would need to be a credible biological explanation of how the estimates derived from the secondary analysis could deviate statistically significantly from the well-known and well-defined test characteristics of the WCS ELISA testing. 

A similar analysis can be performed on ELISA test results for New Brunswick. In 2014, 1486 ELISA tests were ordered in NB (Higgins, personal communication), resulting in 61 positive ELISA tests, 5 of which were confirmed by second-tier western blot testing [33]. While information on the number of patients represented by these 1486 tests, clinical presentation, possible tick exposure, and if timing of the testing after potential tick exposure was appropriate for detection of a serological response is not readily available, it is reasonable to assume that there would have been sufficient suspicion of infection to warrant serological testing and that most tests would represent C6 ELISAs performed through the National Microbiology Laboratory; few health units in New Brunswick performed in-house ELISA testing in 2014. The National Microbiology Laboratory has reported confirming by western blot employing only the formal, strict band criteria defined by the CDC as this testing was done in the context of clinical practice only 5 positive cases of Lyme disease in NB for 2014 from 61 specimens received with first-tier C6 ELISA positivity [33]. Given that the total number of ELISA tests conducted in NB in 2014 is known, as are the numbers confirmed by conventional western blotting, a two-way table can be deduced based on the known test characteristics of C6 ELISA sensitivity (75% overall sensitivity) and specificity (98.5% specificity overall) (Table 3). The analysis demonstrates that the prevalence of Lyme Disease in NB in 2014 is best estimated to have been 3.5% of those being tested, and that 61 positive C6 ELISA tests should be interpreted as reflecting 52 “true” cases of Lyme disease in the province that year. The confirmation of only 5 cases from a population deduced to have 52 cases demonstrates overall sensitivity of the diagnostic methodology of only 9.6% (5/52), or an under-diagnosis magnitude in NB for 2014 of 10.4-fold due to serology alone.

In the face of these discrepancies between the predicted outcomes of well-characterized serological tests and reported results, a report by Wormser et al. (2008) [46], indicating that performance on the C6 ELISA and consequently in two-tiered testing depends on the genotype of the infecting *Borrelia* strain, is significant; sensitivity varied from 66.7%–75% depending on the ribosomal spacer type (RST) genotype. A recent report on testing variability in Canada [33] further illustrates the potential magnitude of *Borrelia burgdorferi* under-diagnosis resulting from inability of the secondary western blot test to confirm infection in those with positive first-tier C6 ELISA results. Analysis of the overall calculated accuracy of two-tier testing in the United States, using sensitivity and specificity characteristics derived from clinical studies, can be shown to be 98.988% compared with accuracy of the single-tier C6 ELISA to be 98.75% [49]. In that context, it is noteworthy that of 2524 Canadian specimens examined by Ogden et al. (2017) [33] in the five years from 2011 to 2015, inclusive, that were C6 ELISA positive, only 557 specimens (22.1%) were confirmed by two-tier western blot analysis. This finding would require either that specificity of the C6 ELISA test is well below the 98.9% that the test is acknowledged to have within a non-endemic area, or that the two-tier confirmatory test using western blot analysis and CDC diagnostic criteria is too unreliably insensitive to be used as a clinical diagnostic tool in Canada.

### 3.3. Estimation of Human Lyme Borreliosis Prevalence from Canine Sentinel Data

Dogs have been used as a sentinel species for sensitive and efficient detection of human Lyme disease risk in the United States [50,51,52,53,54,55,56,57,58,59,60,61,62], Europe [63,64], Asia [65], and Canada [37,38,39,66,67,68,69,70]. Dogs are a valuable sentinel species as they live with humans and serological detection is based on well validated, widely used point-of-care commercial C6 ELISA testing [62,71]. The use of C6 ELISA testing as a stand-alone test avoids false negative results due to *Borrelia* genetic diversity that may not be captured in two-tiered testing [72]. Dogs mount a robust immune response to *Borrelia*, developing higher antibody titers, and antibodies to more VlsE antigens than primates [73], so false negatives and positives are low; the sensitivity and specificity of this testing in dogs has been reported as 98.8% and 100%, respectively, and is not confounded by vaccination induced immunity [71]. Quantitative correlations have been documented between canine *Borrelia* seropositivity and human Lyme disease prevalence [50,52,54,56,62].

**Figure 2:**
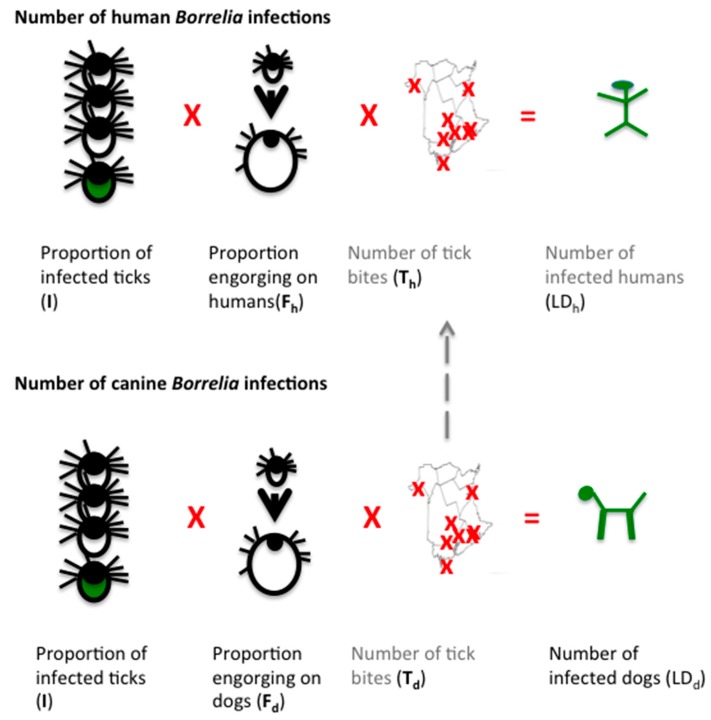
A schematic illustration of the algebraic logic underlying the calculation of human Lyme disease cases from canine sentinel data. *Borrelia* infection (green) requires the bite of an infected tick (green tick) (**I**) and engorgement of that tick for sufficient time to transmit infection (depicted as larger tick) (**F**). Multiplied by the number of tick bites indicated by crosses on a map (**T**), this gives the number of infected individuals (**LD**), depicted by green human and dog stick figures. Both dogs and humans acquire infection in the same manner. Known values (black text) are the proportion of infected tick, typical feeding time for each host and the number of infected dogs in NB. This allows calculation (calculated figures indicated in grey) of the number of tick bites required to produce that many infected dogs (**T_d_**). With adjustment for the relative exposure to ticks (dashed line), the predicted number of human tick bites (**T_h_**) can be calculated and so this formula allows calculation of the predicted number of infected humans (green stick figure) independently of human serological diagnosis and surveillance reporting.

In this work, canine and tick surveillance information is used to assess human Lyme disease prevalence based on the rationale that in order for a human to acquire a *Borrelia* infection, they must be bitten by a tick, that tick must be infected with *Borrelia*, and that tick must feed long enough to transmit the bacteria. Thus, the infection prevalence equals the proportion of infected ticks (**I**), multiplied by the proportion of ticks feeding (engorging) long enough to transmit infection (**F**), multiplied by the number of tick bites (**T**) (Figure 2). As the same rationale applies to dogs, one can use canine seroprevalence data to estimate human infections, which is the rationale underlying the use of dogs as a sentinel species for Lyme disease surveillance. For dogs, the proportion of infected ticks and those feeding for long enough to transmit infection, as well as canine infection prevalence is known, which allows calculation of the remaining variable, the minimum number of tick bites required to produce that number of canine infections. This value can then be used to calculate human infections, once corrected for the relative attractiveness of canine versus human hosts. As this approach relies on extrapolation of canine *Borrelia* seropositivity prevalence, it calculates prevalence rather than incidence.

#### 3.3.1. **I**—Proportion of Infected Ticks

Passive tick surveillance data captures information on both the proportion of infected ticks and tick engorgement. Passive tick surveillance data is available for all Canadian provinces [35]. In NB, this national data is complemented by regional data, which reports an infection rate of 12.3% in 2014 for NB [37].

**I** = 0.123 (1)

#### 3.3.2. **F**—Proportion of Tick Sufficiently Engorged to Transmit Infection

**Figure 3:**
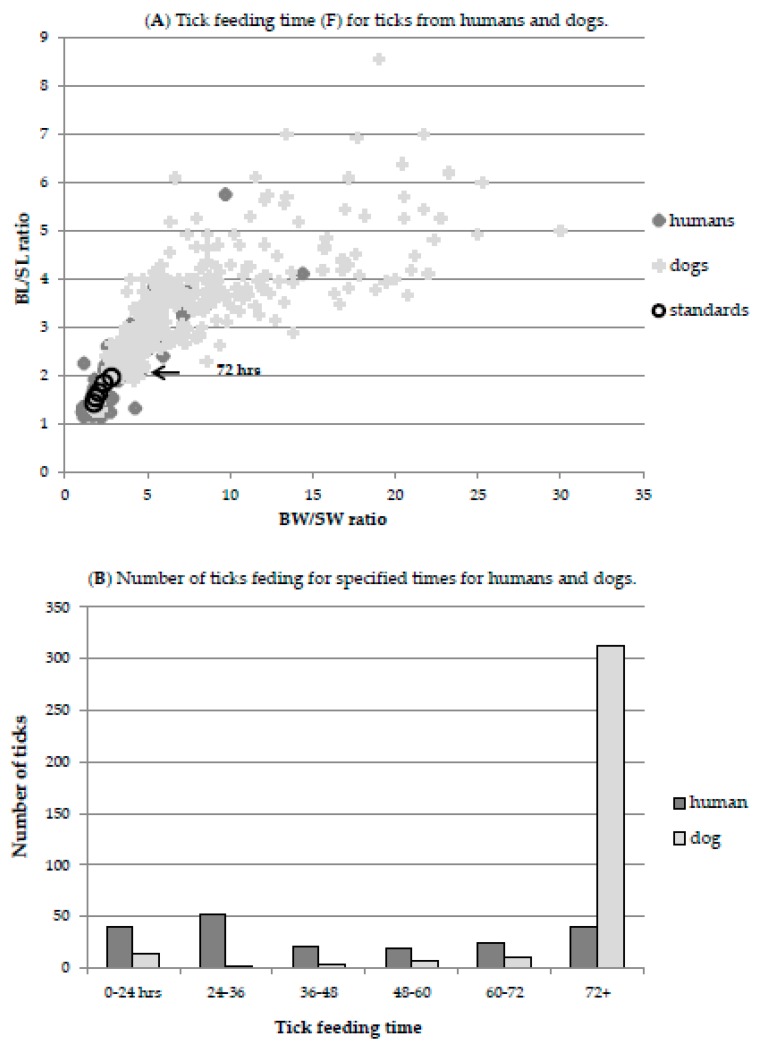
Tick feeding time (F) for ticks from humans and dogs. Tick engorgement indicates duration of feeding, which is correlated with the risk of *Borrelia burgdorferi* transmission. As ticks engorge they expand, however, the relatively inelastic scutum does not expand, providing a means to scale engorgement, as described in the Materials and Methods and by Gray et al. (2005) [31]. The ratio of body (B) to scutum (S) width (BW/SW) and body length (BL) and scutum length (BL/SL) increases with engorgement. The duration of feeding can be mapped onto this function to determine the number of ticks feeding for a given amount of time. (**A**) Dimensions of ticks recovered from humans (dark grey diamond), dogs (light grey cross) and ticks fed for defined times (large black circles) are shown. The defined times are 24, 36, 48, 60 and 72 (indicated by arrow) hrs. (**B**) The number of ticks in each size-defined feeding class for humans (dark grey) and dogs (light grey).

There is vigorous debate about how long a tick must feed to transmit *B. burgdorferi*. Transmission time is confounded by evidence that tick species, developmental stage, bacterial species and strain, host, prior tick feeding, and other factors, all affect transmission time [74,75,76]. From our passive surveillance data (Figure 3), 40/194 (20.6%) ticks from humans had engorged to the extent compatible with 3+ days of feeding. Transmission has been shown to occur at reasonable frequency within 48 h in many studies [74], and 43% had fed for 2+ days but the calculations presented here are based on a conservative requirement of 72 h feeding for transmission. For dogs, 312/348 (90%) ticks from 2014 had engorged to the extent that they must have fed for 3 or more days (95% had fed for 2+ days).

**F_h_** = 0.206 (humans) and **F_d_** = 0.90 (dogs) (2)

#### 3.3.3. **T**—Number of Tick Bites

Calculating the number of tick bites directly is not possible as most go unnoticed, regardless of host [1]. However, tick bites can be extrapolated from canine seropositivity values independently of tick detection as tick bites are reported by seroconversion. Dogs mount a strong immune response to *Borrelia* infection and as the sensitivity and specificity of the commercial C6 ELISA test in dogs is 98.8% and 100%, respectively [71], seropositivity data is robust. Canine seropositivity is known with high certainty in NB from a province-wide high-density canine seropositivity study [37]. As for humans, for a dog to be infected it must have been bitten by an infected tick that was allowed to feed long enough to transmit disease (Figure 2). The relative attractiveness of dogs versus human hosts can be obtained both from passive surveillance data as well as the literature. Together, this can be used to estimate the number of tick bites that would be expected for humans.

The use of canine sentinel data to calculate the number of tick bites allows us to circumvent the intrinsic uncertainty in tick recovery by recording only “productive” tick bites—those resulting in seropositivity. This provides a conservative estimate as it is based on the assumption of one tick per dog. Analysis of our passive tick surveillance data shows that an average of 1.33 ticks recovered per dog. As only one productive tick bite is recorded, additional productive tick bites will not be counted. Additionally, Dibernardo et al. (2014) [35] report that ticks from dogs are more likely to be infected than ticks from humans, presumably as they acquire infection from their host [77]. Although not all ticks become infected when feeding on an infected host, as the canine seroprevalence rate is known, we could adjust the tick infection rate downwards to account for some being secondarily infected by their canine hosts to give a value of 11.6% infected ticks (a 6% decrease in **I**, reflecting 6% canine seroprevalence in NB), requiring more tick bites to produce the observed number of seropositive dogs. However, we again elected to use the more conservative values and did not adjust for canine reservoirs. Additionally, although untreated dogs can remain seropositive for more than a year this is not necessarily true of all infected dogs or treated dogs [78]. Further, the canine seroprevalence value necessarily reflects the shorter lifespan of dogs than humans; the average age of dogs in the study was 5 years [37]. These factors all contribute to an under-estimation of the number of tick bites, **T**, below, so estimation of human *Borrelia* infection prevalence from this data is again conservative.

Bjurman et al. (2016) [37] report 6% of NB dogs were seropositive in 2013/2014 (*n* = 699). A 2014 survey by the Canadian Animal Health Institute [79] found that 34% of Canadian households have a dog. Statistics Canada reports that as of 1 January 2014, the population of NB was estimated at 755,464 [80] and the 2011 census reports an average of 2.3 New Brunswick residents per household [81]. This gives 111,677 dogs in NB, of which 6%, or 6700, would be expected to be *Borrelia*-seropositive. To produce 6700 *Borrelia*-seropositive dogs with a 12.3% of ticks being infected and with 90% feeding for 3+ days (11.07% infection risk per tick), gives an estimated 60,524 tick bites.

**T_d_** = 60,524 tick bites (3)

#### 3.3.4. Extrapolating Dog Tick Bites to Human Tick Bites

Knowing the minimum number of ticks in a region required to produce a given canine seropositivity incidence allows estimation of the risk of tick bites to humans. However, the relative attractiveness of canine to human hosts must be considered. Not only are dogs generally hairier and more pungent than humans, they tend to interact more directly with tick habitats, all factors that increase tick encounters. So to what extent is a tick more likely to end up on a dog relative to a human? This can be determined from the literature or from the return rates from passive surveillance programs.

There are several studies quantifying the correlations between canine and human *Borrelia* infections and when applied to the canine seroprevalence values for New Brunswick, all generate estimates of human seroprevalence appreciably greater than the reported 5 Lyme disease cases reported for 2014 in NB [32] or the cumulative value of 24 Lyme disease cases recorded in NB [32]. Mead et al. (2011) [62] reported that once canine seropositivity was greater than 5%, the median annual human Lyme disease incidence rose sharply, to 24/100,000 and was positively correlated with canine seropositivity. This would predict 181 human Lyme infections annually in NB (24 × population of 755,464/100,000). Similarly, Lindenmayer et al. (1991) [56] found that the ratio between human and canine *Borrelia* infection was 1:6 (14%) in Massachusetts. This ratio encapsulates both the greater likelihood of tick engorgement on dogs versus humans and the greater likelihood of dogs encountering ticks, as a result, it underestimates the relative number of human tick bites as fewer are likely to be productive. If this value were used, one would predict a cumulative total of 6475 cases of human Borrelia infections in NB as of 2014 (6% canine seroprevalence × 14% human to canine seroprevalence × a human population of 755,464). Other studies addressing the correlation between human and canine infection rates also give high estimates for the number of human infections in NB. Eng et al. (1988) [52] reported a 2.6-fold higher infection in dogs relative to humans, in households with a dog. Applied to NB, this would generate a prevalence prediction of 2569 human infections (755,464 population/2.3 people/household = 328,463 households, of which 34%, 111,677, have dogs and risk of infection for an individual in that household is 6%/2.6 or 2.3% = 2569).

An alternate approach to estimate the relative attractiveness of canine and human hosts is to examine the number of ticks recovered from these hosts in passive surveillance. In NB, ticks from both humans and dogs can be submitted for *Borrelia* testing to either the national program or the Mount Allison tick testing program; other alternatives exist but are user-pay so use of these options is negligible where free testing is available. The national tick testing program collects ticks through the healthcare system, hence most ticks from humans are submitted to this program whereas the academic program is directed at ticks contributed by veterinarians, hence ticks from canine hosts are primarily acquired. Between these two programs the majority of detected ticks from humans and dogs can be assumed to be captured. Dibernardo et al. (2014) [35] report that of the ticks received from NB, 187 were from dogs and 123 from humans. In 2014 the Mount Allison tick testing program recovered 429 ticks from dogs and 25 from humans [36]. This gives a total of 616 ticks submitted from dogs and 148 submitted from humans, so ticks were recovered on humans 19% as frequently as on dogs. This value may be conservative; in other years, a higher ratio of human:canine hosts for submitted ticks was recorded [36]. The differential recovery of ticks from dogs versus human hosts is also a consideration; ticks are easier to find on humans versus dogs as evidenced by the larger number of ticks removed before 3 days of feeding from humans than dogs (Figure 3). The proportion of ticks that are not discovered from either host is not readily calculated.

Using the calculated number of canine tick bites, this gives us an estimate of human tick bites equal to 19% of the 60,524 dog tick bites equates to 11,500 human tick bites.

**T_h_** = 11,500 (4)

The information above allows us to calculate the expected number of human *Borrelia* infections (**LD**) in NB in 2014. These would be the product of the infection incidence in ticks (**I**), the proportion of ticks engorging for 3+ days on humans (**F**), and the number of ticks feeding on humans (**T_h_**).

**LD** = **I** × **F** × **T_h_** (5)

Multiplying the infection rate (**I**) of 12.3%, the probability of a tick on a human having fed 3+ days (**F**), 20.6%, and 11,500 human tick bites (**T_h_**), gives an estimate of 291 predicted human infections in NB in 2014. A total of 5 human Lyme disease cases were recorded in NB in 2014, and a cumulative total of 24 Lyme disease cases were recorded from 2010–2014 (prior years had no cases recorded) [32]. This indicates a 291/24 (prevalence) or 291/5 (incidence), 12.1 to 58.2-fold under-detection. The appreciable discrepancy between reported and predicted human infections occurs even though input values were conservative at all stages of the calculations.

## 4. Discussion

These analyses provide three different approaches to estimate the discrepancy between actual and reported Lyme disease infections. The three approaches, comparison of reported Lyme disease incidences in neighboring jurisdictions, evaluation of serological results based on the published sensitivity and specificity metrics for those tests, and canine sentinel studies use different methodologies yet all gave a similar range for under-detection of Lyme disease, with estimates ranging from 12.1 to 58.2-fold (1.7% to 8.3% of all cases detected). In this analysis, the province of New Brunswick was used as a case study, however, the consistent discrepancy between US and Canadian Lyme disease incidences show that the findings are applicable across Canada.

**Figure 4:**
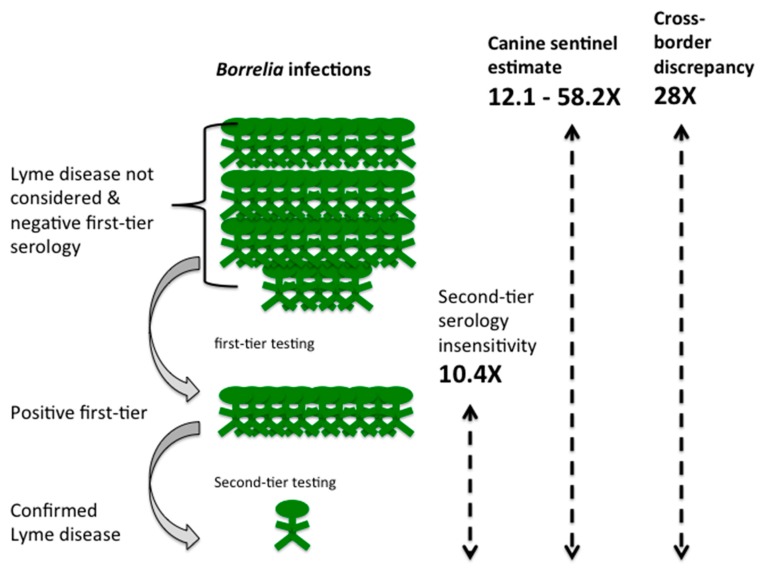
Discrepancy between reported and predicted Lyme disease cases using New Brunswick (NB) as a case study. For every confirmed case recorded (green person, bottom row) there is both a deduced 10.4-fold loss of diagnoses due to failure to obtain positive second-tier serology (9.6% of cases reported; green people, middle row). No cases defined as probable cases, which would include diagnoses of acute Lyme disease without the need for serology, have been reported for NB. Failure to consider Lyme disease as a diagnosis and/or negative first-tier serology results in additional loss of detected infection (green people, top row). Cross-border incidence values indicates that this loss is 28-fold (3.6% of cases detected overall) and analysis of canine sentinel data provides an estimate of 12.1 to 58.2-fold loss (1.7% to 8.3% of total cases detected; the average value is depicted in the figure). Overall, this analysis indicates that for every person with a reported case of Lyme disease, there are approximately 30 other people with Lyme disease excluded by serological insensitivity, failure to consider Lyme disease as a diagnosis or failure to report acute Lyme disease cases.

Under-detection of Lyme disease can arise from diverse causes. These include non-reporting of clinically diagnosed acute Lyme, failure by both patients and healthcare providers to identify acute Lyme, failure to consider Lyme disease as a differential diagnosis in disseminated stages and hence initiate serological testing, and insensitivity of the two-tiered serological testing algorithm to accurately capture infections (Figure 4).

**Under-detection due to serology:** Failure to identify genuine Lyme disease cases due to a false negative in the second tier of the two-tiered serology algorithm is the most easily quantified cause of under-detection. Either a WCS ELISA or an ELISA based on the conserved C6 peptide of the vlsE protein is able to detect a wide range of *Borrelia burgdorferi* strains and *Borrelia* genospecies of the Lyme borreliosis group, with the WCS ELISA having a slightly lower specificity [46,47,49,82]. However, the immunoblot that constitutes the second tier of testing is based on the B31 lab strain of *Borrelia burgdorferi* in most commercial tests. Impaired detection of *Borrelia burgdorferi* strains other than the B31 strain has been described in a number of studies [46,83,84], a problem compounded by the two-tiered testing algorithm not generating a positive result for *Borrelia* species other than *Borrelia burgdorferi* [84,85,86,87]. For example, Wormser [46] et al. have reported only 25% two-tier testing positivity in infections with strains of *Borrelia burgdorferi* with ribosomal spacer type 3 (RST3). This study also reported a number of genotypes based on outer surface protein C (OspC) variability that fail to demonstrate sensitive positive two-tier test results; among 11 individuals infected with OspC type N *Borrelia burgdorferi*, only 5 (45.5%) had positive C6 ELISA and only 1 (9.1%) had positive two-tier testing [46]. This is the third most common *OspC* allele identified in a survey of Canadian *Borrelia* strains [88]. This limitation is very much a concern in the presence of diverse genospecies and strains of *Borrelia*, as would be expected along the coasts of Canada where migratory birds could introduce ticks with different *Borrelia* strains and species from distant sites in Europe, North America, and South America. High genetic diversity of *Borrelia* genospecies and strains in Canada have been reported based on multilocus sequence typing [88] and whole genome sequencing [89] and this has the potential to confound disease prevalence estimates based on serology. Indeed, a comparison of western blot confirmation of positive first tier testing across Canada showed that western blot confirmation was lowest in New Brunswick and on the west coast of the country [33]. This is also consistent with the results of Kadkhoda and Gretchen (2018) [90], who observed that the sensitivity of Canadian second-tier testing was improved from 38% to 61% in physician-confirmed Lyme disease cases by using an alternate approved western blot kit that included both recombinant *Borrelia burgdorferi* B31 and European Lyme antigens. The low rate of second-tier confirmation with conventional testing means that either the C6 ELISA assay as a first-tier test performs with far lower specificity than previously found, or the western blot sensitivity is much lower than expected in this population using the standard commercial kits and strict band definitions. Whether due to geographic strain variability or other factors the reasons and implications warrant further investigation.

The analysis of serological testing presented here has identified that the two-tier test criteria has unreliable sensitivity for its use in seroprevalence estimation, and by extension primary diagnosis. An analysis of testing methodologies [89] has identified that the contemporary first-tier test of the two-tier methodology, the C6 ELISA, has acceptable test characteristics to be employed as a “stand alone” single-tier test, with superior sensitivity compared to two-tier testing and with specificity that remains quite acceptable for clinicians to rely upon as a supportive adjunct for diagnosis. The National Microbiology Laboratory data confirms that of the 2524 C6 ELISA Canadian specimens examined by Ogden et al. (2017) [33] in the five years from 2011 to 2015, only 557 specimens (22.1%) were confirmed by second-tier western blot analysis. If the single-tier C6 ELISA testing methodology were to be accepted as being sufficient to substantiate a diagnosis of Lyme disease in its own right, the data presented here would identify a 4.5-fold magnitude (ratio of C6 ELISA positives to western blot confirmed = 2524/557 = 4.53) of under-reporting due solely to laboratory functions, arbitrary definitions, or testing characteristics. Furthermore, the analysis of the C6 ELISA test shows that its overall sensitivity across all stages of Lyme disease is around 75% to 76.5% [47,89]. Accordingly, those with Lyme disease whose first-tier test was falsely negative could legitimately be deduced to account for approximately an additional one-third the number of those whose tests were positive (25%/75% = 1/3). The resultant estimate of under-diagnosis and under-reporting of Lyme disease incidence in Canada based upon laboratory test performance alone would then be approximately 6-fold (4.5 × 133%), meaning that status quo sensitivity of serological Lyme diagnosis in Canada may be as low as only 1 of every 6 infected patients, or 16.7%.

**Under-detection due to non-reporting of clinical Lyme disease diagnoses:** Public Health Agency of Canada guidelines recommend that acute Lyme be diagnosed clinically and advise that serological testing is not required because it will not be informative before an antibody response is initiated [19]. Although Lyme disease has been a notifiable disease in Canada since 2009, failure to report cases can occur for any number of reasons [91]. Hinckley et al. (2014) [22] and Nelson et al. (2015) [23] quantified the extent of under-reporting of Lyme disease cases in the United States. Many of the reasons are likely to also lead to under-reporting in Canada, with the exception that under-reporting would apply only to clinically diagnosed acute Lyme disease, as serologically confirmed Lyme disease is detected and reported through the National Microbiology Laboratory. Public Health Agency of Canada data for 2016 [92] reports that only 32% of Lyme disease cases are considered “probable cases”, a category that includes clinically identified cases based on the requirement of a physician-observed erythema migrans and exposure to a risk area, as defined by public health agencies. These restrictions would likely lead to significant under-reporting. Zubek (2015) [27] noted that of the 221 Lyme cases diagnosed in British Columbia, only 13 (6%) were reported, whereas Henry et al. (2011) [26] estimated that only 65% of cases were reported, generating under-reporting estimates of 1.5 to 17-fold. While under-reporting of clinically diagnosed acute Lyme disease cases is a problem for surveillance, it is unclear to what extent this affects patient outcomes. Presumably, a diagnosis of acute Lyme disease would be followed by appropriate treatment, although worryingly, Gasmi et al. (2017) [20] report that 49% of Quebec patients identified with a tick bite were treated with single dose prophylaxis more than 72 h after the tick bite, a treatment not recommended as effective [93].

**Under-detection due to failure to consider Lyme disease as a diagnosis and rejection of a Lyme disease diagnosis due to negative first-tier serology:** In addition to the above causes, under-detection will occur where a Lyme disease diagnosis is rejected due to negative first-tier serology and cases where neither patients nor healthcare providers considered Lyme disease as a diagnosis. These considerations apply to both disseminated and acute Lyme cases. Failure of serological testing can result from a number of factors. Serology is not infallible and its reliability is influenced by time since infection, early antibiotic treatment, immunomodulatory and anti-inflammatory therapies, inhibition of class switching, a decline in the vigor of the IgG response over time, as well as other factors, all of which will influence first- and second-tier serology [94,95,96,97,98,99,100]. Additionally, there will be cases where neither patient nor physician considers Lyme disease as a diagnosis. There is insufficient published information available to disentangle the relative contributions of these considerations to the overall loss of recognized Lyme disease cases. However, in 2014, while only 5 cases of Lyme disease were recognized in NB that year, 1486 ELISA tests were requested (Table 3). The number of serology requests in a region with emerging Lyme disease suggests that primary care physicians are considering Lyme disease, a conclusion also supported by a recent survey of New Brunswick Family physicians [21]. However, this same survey did note that only 18% of physicians, presumed to be primarily those in endemic regions, recalled offering a clinical diagnosis of Lyme disease, suggesting a strong reliance on serology [21]. Further, in the province of Quebec during the same time period, Gasmi et al. (2017) [20] report that 55.6% of serology requests were issued before there would have been sufficient time to mount an immune response. If this can be generalized across the country, this could account for a 2-fold loss of detection of Lyme infections. A relative lack of awareness of the possibility of Lyme disease has similarly been documented in the general population; in a comparison of public awareness of Lyme disease in 2012 between the public in Quebec, Canada and an endemic area in Switzerland, only 15% of the survey respondents from Quebec had a good level of knowledge of Lyme disease compared to 51% of respondents from Switzerland [101]. While the relative contribution of failed serology and non-recognition of the possibility of Lyme disease cannot be determined, the combined effect of these factors can be estimated from the difference between the total estimated Lyme disease infections and the under-detection estimated to arise from second-tier serology insensitivity and under-reporting of clinically diagnosed Lyme disease (Figure 4).

The predicted total Lyme disease incidence and prevalence can be estimated by comparing reported Lyme disease incidences between two regions with similar climate, wildlife *Borrelia* hosts, and human populations but differing social and political responses to Lyme disease, in this case, on either side of the US-Canadian border. The discrepancy in cross-border incidence values described here, have been previously described in public health literature [49]. Additionally, we obtained a minimum estimate of Lyme disease prevalence by extrapolating from canine to human infections. The use of dogs as a sentinel species for detection of human risk of Lyme borreliosis is predicated on the understanding that both dogs and humans acquire infection in the same way, through the bite of an infected tick that feeds long enough to transmit infection. While the logic of this premise is unarguable, the input values are drawn from a variety of studies, each with their own strengths and limitations, so all calculations used conservative values. Both approaches gave similar estimates of under-detection.

Our calculations indicate that only approximately 1 of every 6 infected patients for whom laboratory testing is ordered receives a formal diagnosis of Lyme disease. Similarly, reporting of acute Lyme is estimated at 1 of every 6 patients seen; the number of these patients that are appropriately treated is unknown. This implies that for approximately 4 of 6 patients with Lyme infections, the diagnosis is not considered or not substantiated (Figure 4). The combined estimated under-detection ranges from 12.1 to 58.2-fold (1.7% to 8.3%) under-detection. Research is urgently required to identify, quantify, and address the relative contribution of factors leading to under-detection. While this estimate is anchored with data from the province of NB for 2014, there is no reason to assume that the issues leading to under-detection are regionally specific or would have resolved in the intervening period. If so, the 992 Lyme disease cases in Canada reported by surveillance criteria for 2016 [91], would actually range from 8432 to 56,147.

The primary concern arising from this work is the fate of individuals not receiving correct diagnosis and treatment. An increasing number of Canadians seek diagnosis and treatment outside of the Canadian healthcare system [102]. For those who do not receive treatment or receive treatment for another condition, the outcome would not be expected to be positive. The natural course of late or untreated Lyme disease has been abundantly described in the literature [3,4,5,6,7,8,9,10,11,12] and encapsulates a substantial disease burden of chronic pain, fatigue and cognitive or affective impairment, disability, and possible premature death. One implication of this work is that it would be legitimate to initiate investigation into the presence of *Borrelia* infections in patients with a diagnosis of chronic fatigue syndrome/myalgic encephalomyelitis, fibromyalgia, multiple sclerosis, and Parkinsons disease to ascertain if *Borrelia* infections might contribute to these diagnoses. For all these conditions the etiology is unclear, the manifestations overlap with Lyme disease and instances of misdiagnoses are available in the literature. Additional concerns are that the discrepancy between actual and reported Lyme disease cases leads to the perception of the condition as rare; indeed, this discrepancy drives much of the division between communities and public health officials. Finally, it has been estimated that a case of late stage Lyme disease is 230% of the global cost of a case identified in early stage [103]. If, as our analysis suggests, diagnosis is missed and as a result treatment is delayed or denied in the majority of cases, with identified Canadian cases having increased by 700% in the past decade [91], the societal and economic cost implications are contextually enormous.

## 5. Conclusions

Through analysis of published literature and public health databases, this work suggests that many cases of Lyme disease in Canada are, and have been, undetected, unreported, and hence potentially untreated. Development and use of improved methodologies of laboratory testing, for both clinical diagnosis and epidemiologic monitoring of Lyme disease, is urgently needed. Under-estimation of the problem likely results in inadequate allocation of health care and research resources but most importantly, significant health, social and economic costs from impaired health, quality of life, ability to function and to contribute to society, and the attendant personal suffering for those who truly have Lyme disease.

## Figures and Tables

**Table 1:** Comparison between the reported incidence (per 100,000) of Lyme disease in Canadian provinces and the nearest American states.

Province	Canada Incidence		US Incidence		Fold Difference	
	2014	2016	2014	2016	2014	2016
AB	0.05	0.2	0.5 (MT)	1.2 (MT)	10.0	6.0
MB	0.64	3.9	43.43 (ND, MN)	103.47 (ND, MN)	67.9	26.5
ON	0.72	2.7	11.82 (MN, WI, MI, NY)	20.75 (MN, WI, MI, NY)	16.4	7.7
QC	0.21	2.1	20.55 (NY, VT, ME)	31.14 (NY, VT, ME)	97.9	14.8
NB	0.66	1.5	87.9 (ME)	86.4 (ME)	133.2	57.6
NS	5.8	34.4	87.9 (ME)	86.4 (ME)	15	2.5

**Table 2:** Evaluation of WCS ELISA test parameters for seroprevalence of Lyme disease given 215 positive test results in a population of 1855 individual specimens.

ELISA outcome	Positive (Predicted)	Negative (Predicted)	Total (Observed)
WCS ELISA +	88	127	215
WCS ELISA −	6	1634	1640
Total	94	1761	1855
**Parameter**		**Value**	
Prevalence		5.1%	
Sensitivity		93.6%	
Specificity		92.8%	
Positive Predictive Value		40.9%	
Negative Predictive Value		99.6%	
Likelihood ratio (positive)		12.98	
Likelihood ratio (negative)		0.07	

**Table 3:** Deduced Lyme Disease C6 ELISA test outcomes in New Brunswick in 2014.

ELISA outcome	Lyme Disease Present	Lyme Disease Absent	Total
C6 ELISA test positive	39	22	61
C6 ELISA test negative	13	1412	1425
Total	52	1434	1486
**Parameter**		**Value**	
Prevalence		3.5%	
Sensitivity		75.0%	
Specificity		98.5%	
Positive Predicted Value		63.9%	
Negative Predicted Value		99.1%	
Likelihood Ratio (positive)		48.89	
Likelihood Ratio (negative)		0.25	
